# Identification of novel phenotypes in pediatric sepsis based on blood glucose trajectories

**DOI:** 10.3389/fped.2025.1663890

**Published:** 2025-11-07

**Authors:** Ying Wang, Wanqin Song, Shaojun Li, Xianming Xu, Wenjun Liu, Chengjun Liu, Feng Xu, Jing Li

**Affiliations:** 1 Department of Critical Care Medicine, Children’s Hospital of Chongqing Medical University, National Clinical Research Center for Child Health and Disorders, Ministry of Education Key Laboratory of Child Development and Disorders, Chongqing, China; 2 Department of Emergency, Children’s Hospital of Chongqing Medical University, Chongqing, China; 3 Big Data Center for Children’s Medical Care, Children’s Hospital of Chongqing Medical University, Chongqing, China

**Keywords:** sepsis, pediatric, group-based trajectory modeling, longitudinal bloodglucose, phenotype

## Abstract

**Objective:**

Metabolic heterogeneity in sepsis is a critical determinant of prognosis. This study applied group-based trajectory modeling (GBTM) to identify blood glucose trajectory phenotypes in pediatric sepsis and elucidate their associations with clinical outcomes.

**Methods:**

A retrospective cohort study was conducted, enrolling 1,178 pediatric patients diagnosed with sepsis who were admitted to the pediatric intensive care unit of the Children's Hospital of Chongqing Medical University between 2014 and 2022. Dynamic blood glucose data were collected within 72 h of ICU admission, and GBTM was employed to classify trajectory phenotypes. Multivariate logistic regression was used to identify independent predictors of in-hospital mortality. A subgroup analysis focused specifically on patients with septic shock.

**Results:**

The analysis identified four distinct blood glucose trajectory phenotypes: Group 1 (7.3%): Slow-recovery hypoglycemia, predominantly among infants with severe liver injury, coagulopathy, and hyperlactatemia (in-hospital mortality: 13.79%). Group 2 (59.9%): Normoglycemia with minimal organ dysfunction (reference group; mortality: 5.10%). Group 3 (27.7%): Persistent mild hyperglycemia, characterized by elevated inflammatory markers and mild organ injury (mortality: 8.26%). Group 4 (4.9%): Persistent severe hyperglycemia associated with renal impairment and lactate accumulation (mortality: 17.24%). Multivariate analysis revealed Group 4 as an independent risk factor for mortality (aOR = 3.13, 95% CI 1.38–7.07). In the septic shock subgroup, the mortality risks for Group 1 and Group 4 increased by 5.2-fold and 8.28-fold, respectively (both *P* < 0.05).

**Conclusion:**

GBTM effectively stratifies pediatric sepsis into distinct blood glucose trajectory phenotypes. Persistent severe hyperglycemia (Group 4) independently predicts in-hospital mortality, while slow-recovery hypoglycemia (Group 1) indicates a poor prognosis in septic shock. Phenotype-guided interventions are recommended: early insulin therapy (target blood glucose <10 mmol/L) for Group 4 and prophylactic glucose infusion (target >3.8 mmol/L) for Group 1.

## 1 Introduction

Sepsis, a dysregulated host response to infection causing life-threatening organ dysfunction, is fundamentally driven by maladaptive immunity (1). Diagnosed in children using the Phoenix Sepsis Score (≥2 with suspected/confirmed infection) (2, 3), it affects 25 million pediatric patients annually with 22.9% mortality (4). Despite standardized care bundles, 30%–40% of patients respond poorly to therapies (e.g., fluids, antibiotics, vasopressors), and targeted drug trials consistently fail (5, 6). This reflects sepsis' heterogeneity—a syndrome comprising distinct pathophysiological subtypes (7–9).

This heterogeneity involves intertwined mechanisms: dynamic pro-/anti-inflammatory immune shifts, endothelial glycocalyx damage causing microcirculatory failure, and coagulopathy (10, 11). Crucially, metabolic reprogramming is a central driver, where glucose dysregulation strongly correlates with organ injury and death (12–14). While hypoglycemia and glucose variability predict adverse outcomes (15, 16), hyperglycemia's role remains contentious (17). Current pediatric guidelines strongly advise against insulin-driven tight glucose control (≤7.8 mmol/L) but lack evidence-based thresholds (18)—a gap likely arising from studies ignoring metabolic heterogeneity in unstratified cohorts.

Conventional research relies heavily on cross-sectional glucose metrics (e.g., admission or extreme values) (15, 16, 19), failing to capture dynamic temporal adaptations that may define pathophysiological subtypes. Longitudinal trajectory modeling addresses this need. Group-based trajectory modeling (GBTM) clusters biomarker evolution into distinct phenotypes using polynomial functions (20–25). Pioneering work by Bhavani et al. applied GBTM to febrile trajectories, revealing prognostic subtypes and heterogeneous host responses (25, 26)—providing a methodological blueprint for metabolic phenotyping.

We therefore propose a precision medicine strategy: “metabolic phenotyping-guided intervention”. Using GBTM, we identify pathophysiologically meaningful blood glucose trajectory phenotypes, quantifying their slope, curvature, and amplitude. This study addresses: (1) Existence: Do characteristic blood glucose trajectory phenotypes exist in pediatric sepsis? (2) Clinical relevance: Do phenotypes correlate with unique organ injury patterns and outcomes? (3) Translation: Can phenotypes inform individualized glucose management?

## 2 Materials and methods

### 2.1 Study design

This retrospective cohort study included patients admitted to the Pediatric Intensive Care Unit (PICU) at Children's Hospital of Chongqing Medical University (January 1, 2014 – December 31, 2022). As a major regional referral center in Southwest China, the hospital serves a catchment population of approximately 30 million children, which contributed to the diversity of the study sample. The study was approved by the hospital's Scientific Research and Ethics Committee [No.: 2024 Lun Shen (Yan) 334] with waived informed consent due to its retrospective design.

### 2.2 Participants

**Figure 1:**
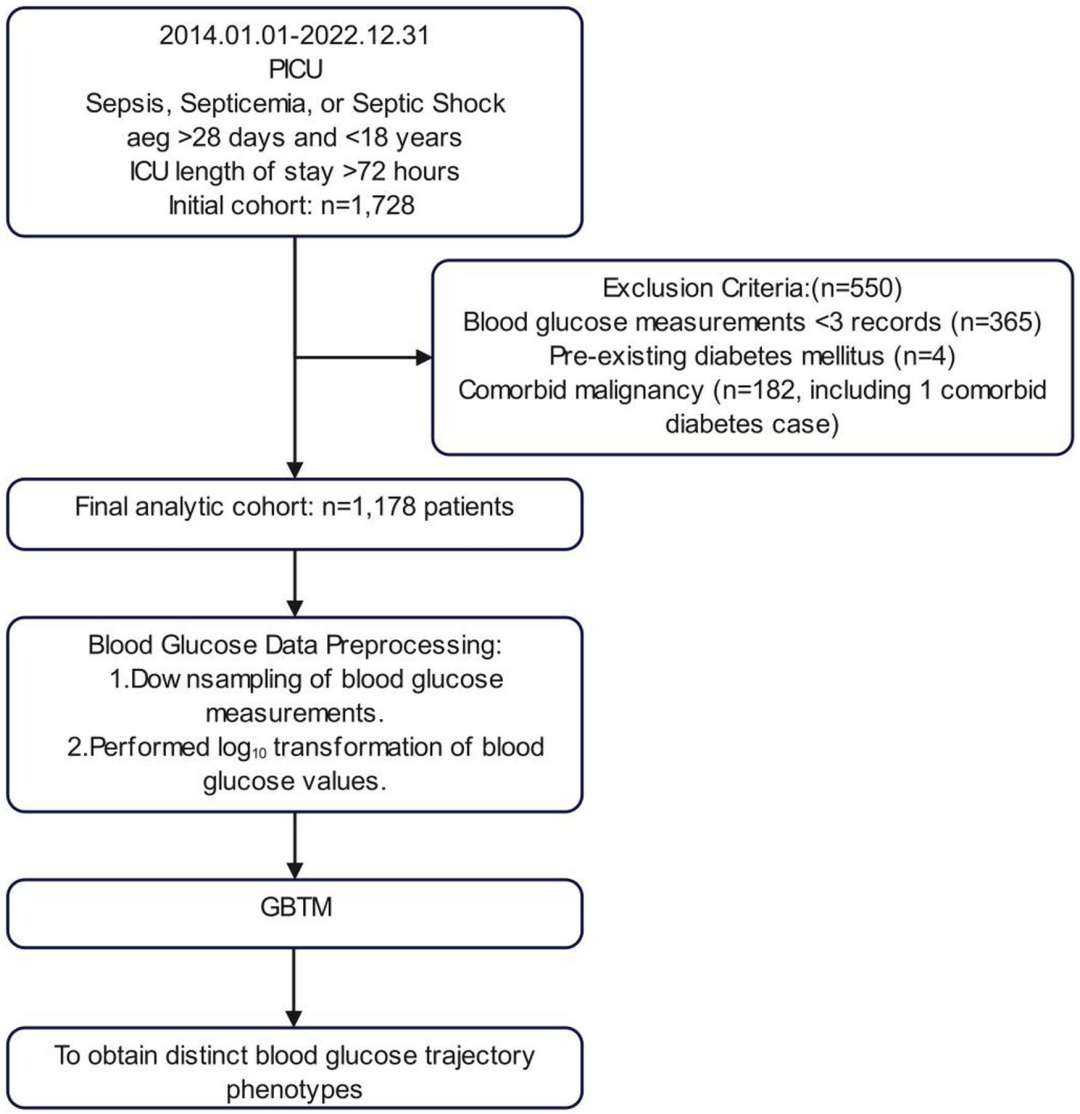
Flowchart of case selection and modeling process.

The inclusion criteria comprised patients with discharge diagnoses of sepsis, bacteremia, or septic shock, aged over 28 days and under 18 years, with a PICU length of stay exceeding 72 h. Exclusion criteria were established for patients with diabetes mellitus or malignancy. To construct trajectory models, participants with fewer than three blood glucose tests within 72 h of PICU admission were excluded, ultimately incorporating 1,178 participants into the analysis (Figure 1).

### 2.3 Data collection

Longitudinal blood glucose measurements (mmol/L) were obtained via arterial blood gas analysis within 72 h of PICU admission. Approximately 75% of the blood gas analyses were conducted using the ABL 90 series analyzer (Radiometer ABL 90 flex; Radiometer, Copenhagen, Denmark), while the remaining analyses were performed using the GEM Premier 3000 analyzer (Instrumentation Laboratory, Bedford, MA) and the Nova Stat Profile® Prime CCS Analyzer (Nova Biomedical, MA, United States). All devices had comparable reference ranges and underwent daily internal/external quality controls. Baseline characteristics included age, sex, traumatic brain injury, and infection site (pulmonary/abdominal/urinary/blood stream/intracranial/skin-mucosal). Laboratory parameters encompassed white blood cell count (WBC), absolute lymphocyte count (ALC), absolute neutrophil count (ANC), lymphocyte percentage, neutrophil percentage, C-reactive protein (CRP), procalcitonin (PCT), fibrinogen (FIB), alanine aminotransferase (ALT), aspartate aminotransferase (AST), total bilirubin (TB), albumin (ALB), lactate dehydrogenase (LDH), platelet count (PLT), international normalized ratio (INR), D-dimer, blood urea nitrogen (BUN), serum creatinine (sCr), and blood lactate (Lac). For parameters measured multiple times within 72 h, the most clinically significant extreme values were recorded, including minimum platelet count, maximum lactate, peak and minimum WBC, and extreme fibrinogen levels. The primary endpoint was in-hospital mortality; secondary endpoints included mechanical ventilation, renal replacement therapy, and septic shock. In this study, the management of dysglycemia in pediatric patients strictly adhered to our institution's standardized clinical pathway (27). Specifically, when a child's blood glucose level consistently exceeded 10 mmol/L, the standard procedure involved immediate discontinuation of all sugar-containing infusions, followed by a 2-hour close observation period. If blood glucose showed a progressive upward trend during this period, an individualized insulin regimen was initiated, accompanied by an increase in blood glucose monitoring frequency to at least once per hour. For patients with hypoglycemia (blood glucose < 2.8 mmol/L), the clinical team promptly administered intravenous glucose infusion as an emergency intervention until blood glucose levels stabilized within the normal range. No missing data existed for baseline characteristics or endpoints. Laboratory parameters (max missing rate: 3.3% for PCT) underwent median imputation.

### 2.4 Statistical analysis

GBTM, a finite mixture model using polynomial time functions, clustered longitudinal blood glucose data (20, 21). To optimize model performance, we implemented several preprocessing steps: (1) For patients with more than 10 glucose measurements within 72 h, we extracted maximum and minimum values at 6-hour intervals, yielding between 3 and 25 data points per patient. (2) Given the significant right skewness of the glucose values, we applied a log10 transformation prior to modeling (Figure 1). To balance model fit and parsimony, we determined trajectory groups by integrating statistical metrics with clinical interpretability. The criteria for model selection included an average posterior probability (AvePP) greater than 70%, closer alignment between posterior probability (Pj) and estimated group probability (πj), lower Bayesian Information Criterion (BIC) values indicating a better fit, higher relative entropy (Ek) values, statistically significant polynomial functions for each trajectory group (*P* < 0.05), and clinically interpretable trajectory shapes. Continuous non-normal variables (Shapiro–Wilk) reported as median [IQR]; compared via Mann–Whitney U (2-group) or Kruskal–Wallis H (≥3 groups). Categorical variables reported as *n* (%); compared via *χ*^2^ or Fisher's exact test (expected counts <5). Bonferroni correction applied for significant multi-group comparisons. Multivariate logistic regression (stepwise) identified independent predictors. Discriminative power assessed by ROC-AUC (95% CI).

Trajectory modeling was performed using the SAS software package (version 9.4) with the PROC TRAJ application, while all other statistical analyses were carried out using R software (version 4.3.0). Two-tailed tests were employed throughout the study, with a significance level set at *α* = 0.05; *P* values less than 0.05 were considered statistically significant.

## 3 Results

### 3.1 Blood glucose trajectory phenotypes

**Figure 2:**
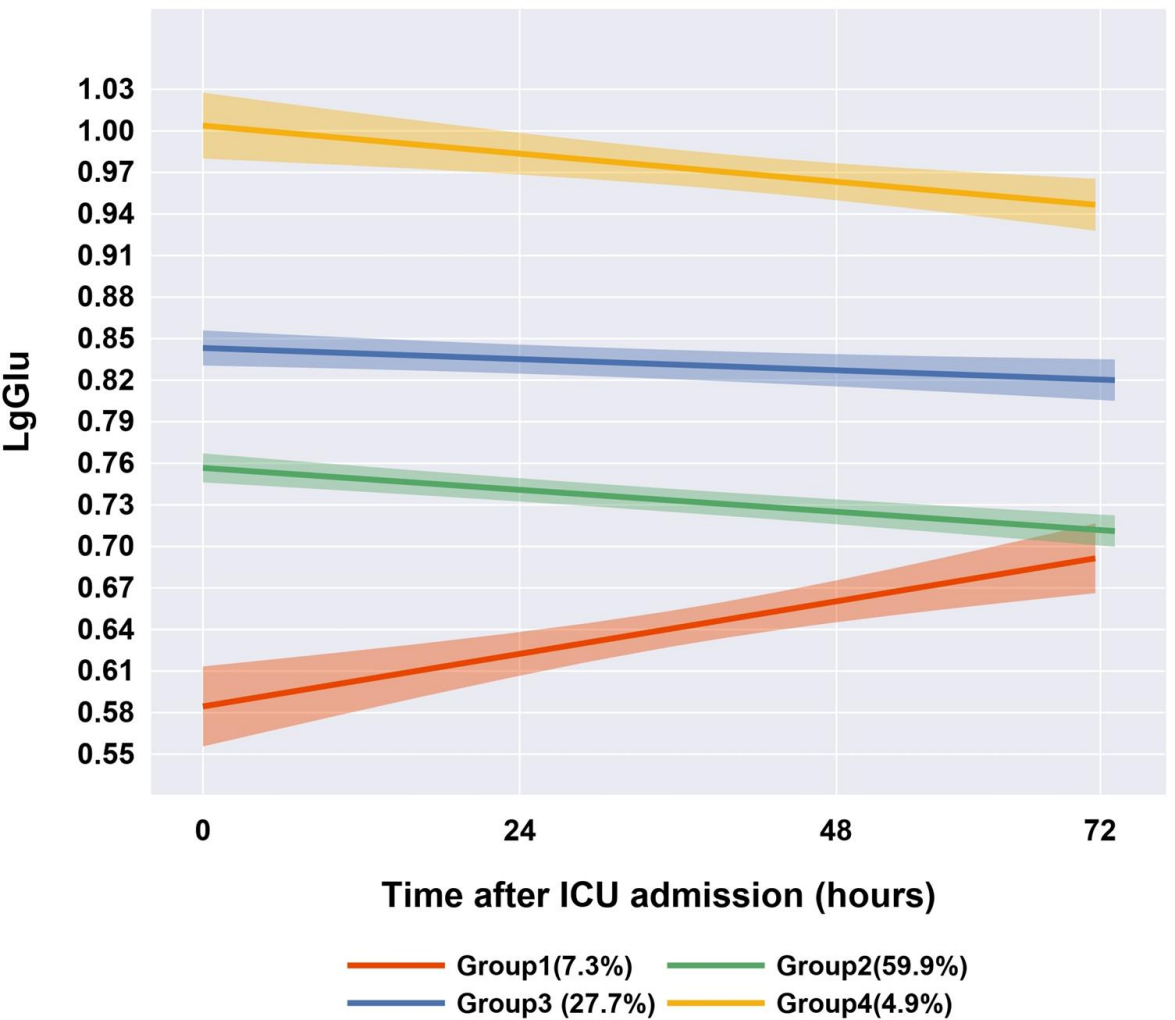
Four-group blood glucose trajectories of the best-fitting group trajectory model (*n* = 1,178).

We initially fitted linear, quadratic, and cubic polynomial functions to identify 1–5 trajectory groups. The optimal model revealed four distinct trajectory phenotypes, determined through a comprehensive evaluation of the BIC, the Ek and clinical interpretability (Supplementary Figure S1 and Tables S1–S4): Group 1 (Slow-Recovery Hypoglycemia): Characterized by initial hypoglycemia (median 3.8 mmol/L) with gradual normalization, observed in 87 cases (7.3%). Group 2 (Normoglycemia): Exhibited stable glucose levels (median 5.7 mmol/L) throughout the 72 h of PICU admission, representing the largest subgroup with 706 cases (59.9%). Group 3 (Persistent Mild Hyperglycemia): Demonstrated sustained mild hyperglycemia (median 7.0 mmol/L) in 327 cases (27.7%). Group 4 (Persistent Severe Hyperglycemia): Displayed prolonged severe hyperglycemia (median 10.1 mmol/L) in 58 cases (4.9%) (Figure 2). This GBTM analysis stratified pediatric sepsis patients into four distinct blood glucose trajectory phenotypes.

### 3.2 Intergroup differences in clinical characteristics

Age distribution differed significantly (*P* < 0.001): Group 1 had highest infant proportion (68.97%) vs. Group 2 (reference); Groups 3–4 were predominantly >1 year. Group 3 showed highest traumatic brain injury rate (2.75%, *P* < 0.05). No sex/infection site differences (Table 1).

**Table 1:** Comparison of baseline characteristics among the four phenotypes.

Variables	Total (*n* = 1,178)	Group1 (*n* = 87)	Group2 (*n* = 706)	Group3 (*n* = 327)	Group4 (*n* = 58)	Statistic	*P*
Age,(%)						*χ*^2^ = 113.61	<.001*
≦1 year	482 (40.92)	60^c^ (68.97)	342^a^ (48.44)	75^b^ (22.94)	5^b^ (8.62)		
>1 year	696 (59.08)	27^c^ (31.03)	364^a^ (51.56)	252^b^ (77.06)	53^b^(91.38)		
Sex(male),(%)	695 (59.00)	54 (62.07)	416 (58.92)	197 (60.24)	28 (48.28)	χ^2^ = 3.31	0.347
TBI,(%)	12 (1.02)	0^ab^ (0.00)	3^a^ (0.42)	9^b^(2.75)	0^ab^ (0.00)	-	0.010*
Infection sites,(%)							
Pulmonary	986 (83.70)	74 (85.06)	598 (84.70)	266 (81.35)	48 (82.76)	χ^2^ = 2.00	0.572
Abdominal	190 (16.13)	21 (24.14)	104 (14.73)	58 (17.74)	7 (12.07)	χ^2^ = 6.48	0.091
Intracranial	288 (24.45)	22^ab^ (25.29)	198^b^ (28.05)	53^a^ (16.21)	15^ab^ (25.86)	χ^2^ = 17.06	<.001*
Urinary tract	23 (1.95)	0 (0.00)	15 (2.12)	8 (2.45)	0 (0.00)	-	0.479
Bloodstream	292 (24.79)	22 (25.29)	174 (24.65)	86 (26.30)	10 (17.24)	χ^2^ = 2.19	0.534
Skin/mucosal	61 (5.18)	4 (4.60)	37 (5.24)	17 (5.20)	3 (5.17)	–	1.000

TBI,traumatic brain injury; χ^2^: Chi-square test, -: Fisher exact.

* Values marked in bold indicate statistical significance (*P* < 0.05).

a,b,c Significant differences (Bonferroni-corrected *P* < 0.05).

Laboratory parameters revealed marked differences in systemic inflammation, hepatic and renal function, coagulation profiles, and lactate metabolism (Table 2). Group 2, characterized by normoglycemia, exhibited the most favorable physiological status, displaying moderate inflammation, minimal organ injury, normal coagulation, and the lowest lactate levels. In contrast, Group 1 demonstrated a distinct pathological pattern, characterized by attenuated inflammatory responses coupled with severe hepatic dysfunction and coagulopathy, presenting the lowest fibrinogen levels and platelet counts among all groups (*P* < 0.001). Group 3 was marked by systemic hyperinflammation, with significantly elevated inflammatory markers. Conversely, Group 4 mirrored the inflammatory profile of Group 3 but exhibited more pronounced renal impairment. Blood lactate levels followed a gradient: Group 2 < Group 3 < Group 1/Group 4 (*P* < 0.001). Visual representations of the laboratory parameters are illustrated in Supplementary Figure S2.

**Table 2:** Comparison of laboratory parameters among the four phenotypes.

Variables	Total (*n* = 1,178)	Group1 (*n* = 87)	Group2 (*n* = 706)	Group3 (*n* = 327)	Group4 (*n* = 58)	Statistic	*P*
WBC_min (×10^9^/L)	7.55 (4.30, 11.79)	5.79^a^ (3.45,9.71)	7.28^ab^ (4.49,11.45)	8.18^b^ (4.25,12.47)	8.25^ab^ (4.64,12.87)	χ^2^ = 8.82^e^	**0.032** *
ALC (×10^9^/L)	1.44 (0.78, 2.49)	1.61^ac^ (1.02,3.10)	1.65^a^ (0.86,2.66)	1.18^b^ (0.66,1.94)	1.02^b^ (0.63,1.76)	χ^2^ = 42.75^e^	**<.001** *
ANC (×10^9^/L)	4.86 (2.32, 8.54)	2.91^c^ (1.65,5.81)	4.49^a^ (2.28,7.99)	6.15^b^ (2.83,9.79)	6.01^ab^ (3.28,10.93)	χ^2^ = 28.79^e^	**<.001** *
Neut%	0.82 (0.70, 0.95)	0.73^a^ (0.60,0.82)	0.81^b^ (0.67,0.94)	0.88^c^ (0.75,44.90)	0.88^c^ (0.83,60.97)	χ^2^ = 57.51^e^	**<.001** *
PCT (ng/ml)	5.20 (1.13, 27.57)	5.87 (1.47,30.00)	5.20 (0.94,26.12)	5.20 (1.73,28.34)	5.20 (1.05,11.18)	χ^2^ = 5.20^e^	0.158
CRP (mg/L)	35.00 (11.00, 77.86)	26.00^ac^ (8.00,66.06)	32.00^a^ (9.04,73.00)	45.00^b^ (18.52,90.00)	27.00^abc^ (10.00,74.00)	χ^2^ = 16.70^e^	**<.001** *
FIB_max (g/L)	2.72 (1.72, 4.29)	1.58^a^ (1.08,2.82)	2.65^b^ (1.74,4.24)	3.07^c^ (1.98,4.65)	2.78^bc^ (1.86,4.54)	χ^2^ = 53.61^e^	**<.001** *
PLT (×10^9^/L)	155.50 (57.25, 288.75)	62.00^c^ (22.50,197.00)	175.00^a^ (71.25,318.50)	146.00^b^ (50.50,255.00)	136.00^abc^ (57.00,206.50)	χ^2^ = 33.43^e^	**<.001** *
D-dimer (mg/L)	6.22 (2.00, 12.83)	8.16^ab^ (2.58,14.30)	5.36^a^ (1.69,11.83)	6.58^b^ (2.35,14.32)	6.98^ab^ (2.21,12.68)	χ^2^ = 13.41^e^	**0.004** *
FIB_min (g/L)	2.11 (1.25, 3.47)	1.06^a^ (0.55,1.60)	2.17^b^ (1.38,3.52)	2.24^b^ (1.28,3.90)	2.07^b^ (1.25,3.27)	χ^2^ = 63.42^e^	**<.001** *
INR	1.25 (1.08, 1.62)	1.79^a^ (1.24,2.73)	1.20^b^ (1.04,1.50)	1.31^c^ (1.11,1.62)	1.33^bc^ (1.10,1.60)	χ^2^ = 63.90^e^	**<.001** *
ALT (U/L)	47.80 (30.90, 120.07)	106.00^a^ (51.15,459.45)	44.20^b^ (30.00,98.77)	44.90^b^ (29.00,111.05)	75.90^ab^ (36.40,195.05)	χ^2^ = 42.28^e^	**<.001** *
AST (U/L)	81.15 (41.85, 234.83)	174.30^a^ (54.80,1,179.75)	73.30^b^ (41.00,189.72)	79.30^b^ (40.20,232.65)	144.40^ab^ (43.82,424.85)	χ^2^ = 28.91^e^	**<.001** *
TB (umol/L)	10.00 (4.40, 23.50)	23.90^c^ (7.70,100.10)	8.15^a^ (4.00,18.60)	10.80^b^(5.20,26.45)	14.55^bc^ (10.15,36.20)	χ^2^ = 57.64^e^	**<.001** *
ALB (g/L)	27.20 (23.30, 31.20)	25.20^a^ (20.35,28.20)	27.50^b^ (23.83,31.70)	26.60^b^ (22.85,31.05)	29.10^b^ (24.47,32.25)	χ^2^ = 25.03^e^	**<.001** *
LDH (U/L)	472.15 (299.00, 1,097.58)	874.00^c^ (325.85,3375.20)	442.05^a^ (289.25,949.25)	490.00^ab^ (304.20,1116.95)	614.70^bc^ (394.65,1454.83)	χ^2^ = 19.98^e^	**<.001** *
BUN (mmol/L)	4.81 (3.00, 8.50)	6.10^b^ (3.69,10.18)	4.36^a^ (2.64,7.41)	5.50^b^ (3.38,9.71)	7.79^b^ (4.35,12.13)	χ^2^ = 37.14^e^	**<.001** *
sCr (umol/L)	36.00 (24.00, 64.57)	41.80^bc^ (26.50,81.70)	31.35^a^ (22.83,52.98)	42.00^b^ (27.95,78.40)	57.60^c^ (39.67,95.15)	χ^2^ = 54.18^e^	**<.001** *
Lac (mmol/L)	1.80 (1.20, 3.18)	2.70^c^ (1.75,6.95)	1.60^a^ (1.10,2.60)	2.00^b^ (1.40,3.80)	3.10^c^ (2.00,5.30)	χ^2^ = 81.91^e^	**<.001** *

Notes: Data are median (IQR).

abc Significant differences (Bonferroni-corrected *P* < 0.05).

e Kruskal-waills test.

* Values marked in bold indicate statistical significance (*P* < 0.05). WBC_min, minimum white blood cell count; ALC, absolute lymphocyte count; ANC, absolute neutrophil count; Neut%, neutrophil percentage; PCT, procalcitonin; CRP, C-reactive protein; FIB_max, maximum fibrinogen; FIB_min, minimum fibrinogen; PLT, platelet count; INR, international normalized ratio; ALT, alanine aminotransferase; AST, aspartate aminotransferase; TB, total bilirubin; ALB, albumin; LDH, lactate dehydrogenase; BUN, blood urea nitrogen; sCr, serum creatinine; Lac, blood lactate.

Among 1,178 patients, 85 experienced in-hospital mortality, resulting in an overall mortality rate of 7.22%. Mortality elevated in Group 1 (13.79%) and Group 4 (17.24%) vs. Group 2 (5.10%, *P* < 0.001). Mechanical ventilation/renal replacement differed (*P* < 0.05); septic shock incidence was comparable (Table 3).

**Table 3:** Comparison of outcomes and treatment conditions among the four phenotypes.

Variables	Total (*n* = 1,178)	Group1 (*n* = 87)	Group2 (*n* = 706)	Group3 (*n* = 327)	Group4 (*n* = 58)	Statistic	*P*
In-hospital mortality(%)	85 (7.22)	12^b^ (13.79)	36^a^(5.10)	27^ab^(8.26)	10^b^(17.24)	χ^2^ = 19.58	<.001*
Septic shock(%)	208 (17.66)	18 (20.69)	126 (17.85)	55 (16.82)	9 (15.52)	χ^2^ = 0.91	0.823
Mechanical ventilation(%)	868 (73.68)	64 (73.56)	499 (70.68)	256 (78.29)	49 (84.48)	χ^2^ = 10.35	0.016*
Renal replacement therapy(%)	145 (12.31)	15^ab^ (17.24)	63^b^ (8.92)	56^a^ (17.13)	11^ab^ (18.97)	χ^2^ = 18.87	<.001*

χ^2^: Chi-square test, -: Fisher exact; ^abc^Significant differences (Bonferroni-corrected *P* < 0.05); *Values marked in bold indicate statistical significance (*P* < 0.05).

### 3.3 Risk factors for in-hospital mortality in sepsis

Univariate logistic regression was conducted to evaluate the associations between candidate variables and in-hospital mortality. The variables analyzed included: (1) Demographics: age and sex; (2) Clinical features: blood glucose trajectory phenotypes, septic shock, mechanical ventilation, and renal replacement therapy; (3) Laboratory parameters: complete blood count, coagulation profile, hepatic and renal function, and lactate levels. Using Group 2 (normoglycemia) as the reference, both Group 1 (odds ratio [OR] 2.98, 95% confidence interval [CI] 1.49–5.97; *P* = 0.002) and Group 4 (OR 3.88, 95% CI 1.81–8.29; *P* < 0.001) demonstrated significantly elevated mortality risks (Supplementary Table S5). Additional significant predictors included septic shock, renal replacement therapy, decreased PLT, Hb, and FIB, prolonged INR, and increased levels of D-dimer, AST, TB, BUN, and Lac.

In the multivariate logistic regression analysis, variables with a *p*-value less than 0.05 from the univariate analysis were included in the multivariate logistic regression model, employing bidirectional stepwise regression for variable selection. The final model identified five independent predictors: blood glucose trajectory phenotypes, septic shock, renal replacement therapy,TB, and Lac (Table 4). After adjusting for confounding factors, blood glucose trajectory phenotypes remained significantly associated with in-hospital mortality. Compared to Group 2, Group 4 exhibited a 3.13-fold increase in mortality risk [adjusted odds ratio (aOR) 3.13, 95% CI 1.38–7.07; *P* = 0.006]. The multivariate model showed good fit (Hosmer-Lemeshow *P* = 0.589), no collinearity [variance inflation factors (VIF) < 10; Supplementary Table S6], and moderate discrimination (AUC 0.78, 95% CI 0.72–0.84; Supplementary Figure S3).

**Table 4:** Multivariate logistic regression analysis of risk factors for in-hospital mortality in sepsis patients.

Variables	*β*	S.E	Z	*P*	aOR (95%CI)
Intercept	−3.77	0.23	−16.48	**<**.001*	0.02 (0.01∼0.04)
Trajectory phenotypes					
Group2					1.00 (Reference)
Group1	0.42	0.41	1.03	0.303	1.53 (0.68∼3.42)
Group3	0.31	0.28	1.11	0.265	1.37 (0.79∼2.38)
Group4	1.14	0.42	2.74	**0**.006*	3.13 (1.38∼7.07)
Septic shock					
no					1.00 (Reference)
yes	0.61	0.28	2.20	**0**.028*	1.84 (1.07∼3.18)
Renal replacement therapy					
No					1.00 (Reference)
yes	1.29	0.28	4.60	**<**.001*	3.62 (2.09∼6.25)
TB (umol/L)	0.01	0.00	2.49	**0**.013*	1.01 (1.01∼1.01)
Lac (mmol/L)	0.08	0.03	3.10	**0**.002*	1.09 (1.03∼1.15)

aOR, adjusted Odds Ratio; CI, Confidence Interval; TB, total bilirubin; Lac, blood lactate.

* Values marked in bold indicate statistical significance (*P* < 0.05).

### 3.4 Subgroup analysis

Among 208 septic shock patients (from 1,178 sepsis cases), multivariate analysis identified four independent mortality predictors: blood glucose trajectory phenotypes (using Group 2 as the reference: Group 1: aOR 5.20, 95% CI 1.27–21.28; *P* = 0.022; Group 4: aOR 8.28, 95% CI 1.47–46.55; *P* = 0.016), renal replacement therapy, elevated BUN and Lac. The model demonstrated high clinical predictive value, with an AUC of 0.87 (95% CI 0.80–0.93) (Supplementary Table S7 and Figure S4).

### 3.5 Robust analysis

**Figure 3:**
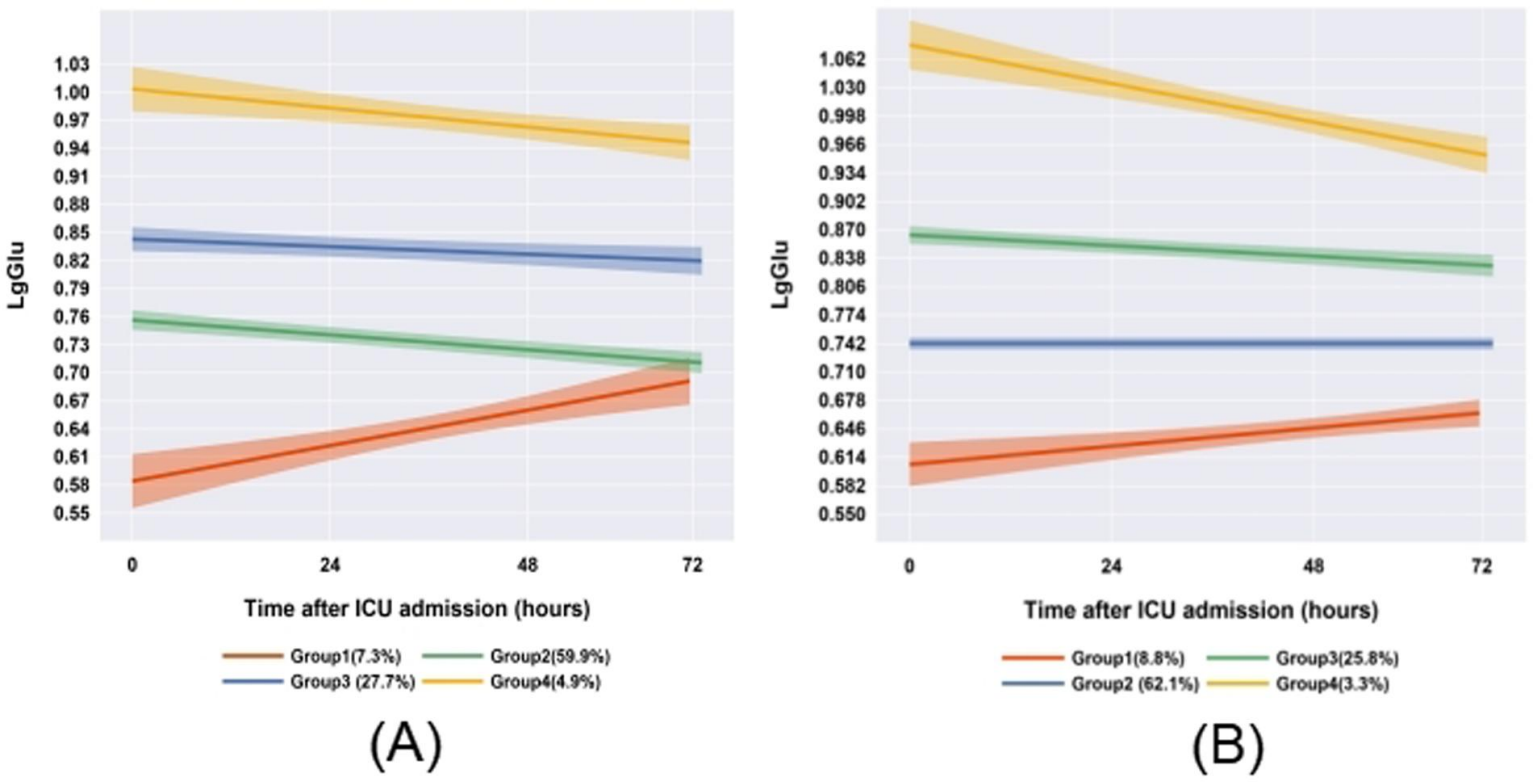
Comparison of glucose trajectories between comorbidity subgroups and the overall cohort (**A**: original cohort; **B**: patients with diabetes/cancer).

To evaluate the stability of blood glucose trajectory phenotypes in patients with comorbidities, we conducted a subgroup analysis of 185 sepsis patients with diabetes or malignancy using GBTM. The expanded cohort consisted of 1,363 patients. Key findings include: (1) retention of the four primary glucose trajectory phenotypes; (2) consistent glucose trends vs. original cohort (*n* = 1,178; Figure 3). This validates phenotype robustness across comorbidities.

## 4 Discussion

Using GBTM, we analyzed longitudinal blood glucose data from 1,178 pediatric sepsis patients during their first 72 h in PICU. We identified four distinct blood glucose trajectory phenotypes: Group 1 (slow-recovery hypoglycemia), Group 2 (normoglycemia), Group 3 (persistent mild hyperglycemia), and Group 4 (persistent severe hyperglycemia). These phenotypes demonstrated significant associations with clinical outcomes. Multivariate regression analysis revealed that Group 4 was an independent risk factor for in-hospital mortality within the overall sepsis cohort (aOR 3.13, 95% CI 1.38–7.07). Notably, in the septic shock subgroup, both Group 1 (aOR 5.20, 95% CI 1.27–21.28) and Group 4 (aOR 8.28, 95% CI 1.47–46.55) exhibited the highest mortality risks. Furthermore, blood glucose trajectories correlated with specific patterns of organ dysfunction: Group 1 was characterized by severe hepatic injury combined with coagulopathy, while Group 4 showed prominent renal impairment. Both Groups 1 and 4 had significantly higher lactate levels compared to Groups 2 and 3 (*P* < 0.001).

### 4.1 Association between blood glucose and prognosis in pediatric sepsis

In pediatric sepsis, early stress hyperglycemia (SHG) may initially support immune cell metabolism via inflammatory/endocrine pathways (13, 17, 28, 29). However, sustained hyperglycemia predicts adverse outcomes (30). Meta-analyses confirm nonlinear mortality risk: U-shaped (diabetic) and J-shaped (non-diabetic) curves, where persistent hyperglycemia beyond optimal thresholds increases mortality (31). Our trajectory analysis validates this, identifying persistent severe hyperglycemia (Group 4; median 10.1 mmol/L) as an independent predictor of in-hospital mortality. Notably, hypoglycemia risk was context-dependent: Slow-recovery hypoglycemia (Group 1) showed nonsignificant mortality risk overall (*P* > 0.05), potentially due to exclusion of early fatal hypoglycemia cases (ICU stay <72 h exclusion). However, in septic shock, Group 1 had critically elevated mortality (aOR=5.20), underscoring its threat in high-risk subgroups.

### 4.2 Blood glucose and organ dysfunction

Group 1 (slow-recovery hypoglycemia) exhibited significantly elevated biomarkers of hepatic injury, potentially linked to the“hepatogenic energy crisis”hypothesis (32). Persistent hypoglycemia may deplete glycogen and impair mitochondrial function, reducing lactate clearance. A vicious cycle ensues: (1) Liver injury suppresses the mRNA expression of phosphoenolpyruvate carboxykinase (PEPCK), limiting gluconeogenesis (33); (2) Decreased hepatic insulin-degrading enzyme (IDE) activity prolongs the half-life of insulin in peripheral circulation (34), worsening dysregulation. Associated coagulopathy (prolonged INR, hypofibrinogenemia and thrombocytopenia) reflects impaired synthetic function (35).

In Group 4 (persistent severe hyperglycemia), serum creatinine levels were significantly elevated compared to other phenotypes, indicating that sepsis-associated hyperglycemia may induce acute kidney injury (AKI) through metabolic toxicity. Hyperglycemia upregulates the expression of sodium-glucose cotransporter 2 (SGLT2) in renal tubules, causing glucose overload and mitochondrial stress—consistent with tubular vacuolization/fibrosis. SGLT2 inhibitors (e.g., dapagliflozin) mitigate this in sepsis-AKI models (36).

In our study, all abnormal phenotypes showed elevated lactate, indicating disrupted energy homeostasis. Sepsis impairs the tricarboxylic acid cycle (TAC), shunting pyruvate to lactate. Hyperglycemia suppresses TCA enzymes (e.g., *α*-ketoglutarate dehydrogenase) (37), while lactate accumulation inhibits gluconeogenesis (38, 39), creating a glucose-lactate vicious cycle.

Our study suggests a potential phenotype-specific interaction between glucose dysregulation and immune dysfunction in sepsis. Hyperglycemic phenotypes (Groups 3 and 4) exhibited enhanced activation of myeloid cells alongside significantly reduced absolute lymphocyte counts, a pattern consistent with CD8+ T cell exhaustion during the immune paralysis phase of sepsis. However, the causal relationship between glucose fluctuations and these immune alterations necessitates validation through expanded immunophenotyping, which should include interleukin-6 (IL-6) levels, monocyte human leukocyte antigen DR (mHLA-DR) expression, regulatory T cell ratio (Treg%), Th1/Th2 cytokine balance, and pathogen-specific antibody titers (40).

### 4.3 Clinical implications of blood glucose trajectories

Optimizing glycemic control in pediatric sepsis remains challenging. Moderate stress hyperglycemia may support immunity, but sustained levels ≥10 mmol/L increase multiorgan dysfunction and mortality (13, 41, 42). Tight control mitigates metabolic harm but risks iatrogenic hypoglycemia (41). Our trajectory analysis identifies two high-risk phenotypes: Group 4 (persistent severe hyperglycemia; median 10.1 mmol/L): highest mortality overall; Group 1 (slow-recovery hypoglycemia; nadir 3.8 mmol/L): critically elevated mortality in septic shock (Supplementary Table S4). Aligning with international guidelines (18, 27, 43–45), we propose a dual-threshold framework: Upper: 10.0 mmol/L (triggering insulin therapy) and Lower: 3.8 mmol/L (activating glucose infusion protocols).

### 4.4 Innovations and limitations

This study advances pediatric sepsis research by using GBTM to link glucose trajectories (vs. isolated measurements) with outcomes. Our key innovations include: (1) the identification of both absolute glucose abnormalities (e.g., hypoglycemia in Group 1, hyperglycemia in Groups 3 and 4) and dynamic patterns (e.g., sustained hyperglycemic plateaus in Group 4); (2) the mitigation of static measurement biases inherent to traditional approaches. However, several limitations warrant attention: (1) As all participants were recruited from a single tertiary pediatric center, our findings may reflect region-specific clinical practices. Generalizability to other settings warrants validation; (2) the lack of model validation means that the GBTM-derived phenotypes require internal and external validation using datasets such as the Medical Information Mart for Intensive Care (MIMIC) database; (3) regarding outcome scope, the inclusion of 28-day mortality and functional recovery metrics would enhance the phenotypic prognostic interpretation.

## 5 Conclusion

This study successfully identified four characteristic blood glucose trajectory phenotypes in children with sepsis using GBTM: Group 1 as the slow recovery from hypoglycemia type, Group 2 as the normoglycemic type, Group 3 as the persistent mild hyperglycemia type, and Group 4 as the persistent severe hyperglycemia type. It ultimately revealed the association between blood glucose trajectories and organ dysfunction, as well as clinical prognosis in children with sepsis, thereby providing a new theoretical basis and practical framework for precise blood glucose management. Future multi-center studies are essential to confirm the generalizability of these phenotypes.

## Data Availability

The datasets presented in this study can be found in online repositories. The names of the repository/repositories and accession number(s) can be found in the article/Supplementary Material.
