# Supplementary material for: Identification of novel phenotypes in pediatric sepsis based on blood glucose trajectories
Source: Front Pediatr. 2025 Nov 7;13:1663890. doi: 10.3389/fped.2025.1663890 (PMC12634563; doi:10.3389/fped.2025.1663890)
Supplement: Supplementary file 1 [file Datasheet1.docx]

**Figure S1. Blood glucose trajectories across trajectory models**

Groups 1 to 5 represent 1–5 trajectory groups modeled by GBTM, respectively; and the optimal model is four distinct trajectory phenotypes.


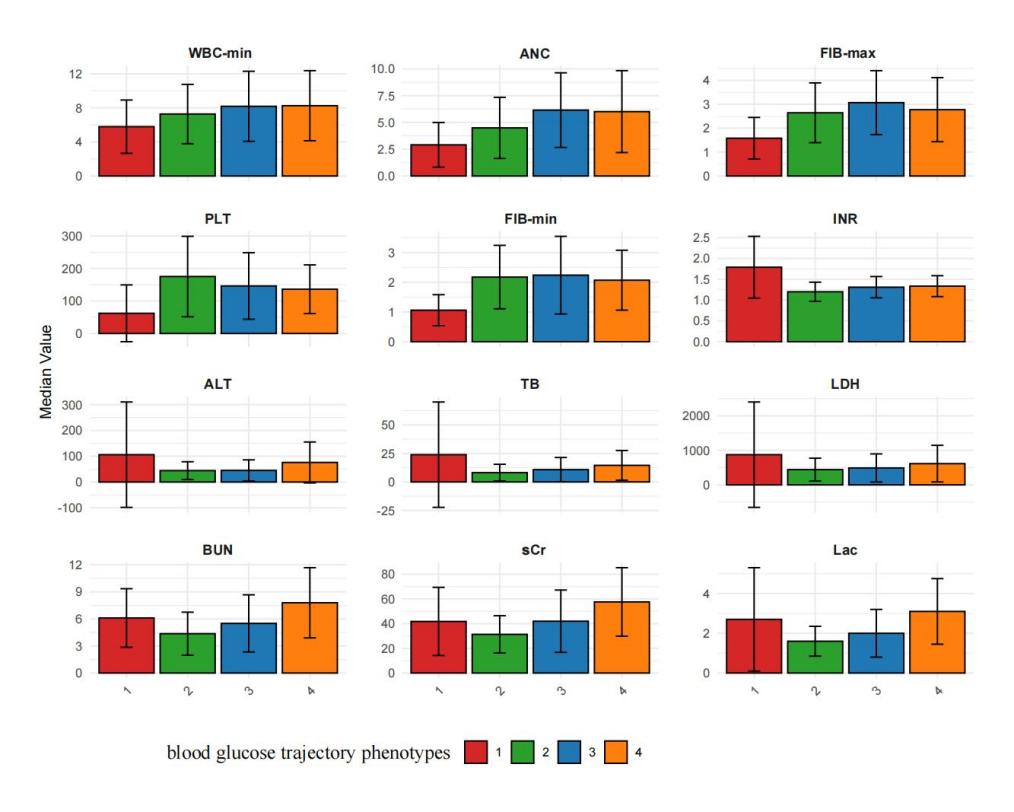


**Figure S2. Clinical parameters by the four phenotypes (faceted bars)**

WBC_min, minimum white blood cell count,×10^9^/L; ANC, absolute neutrophil count,×10^9^/L; FIB_max, maximum fibrinogen, g/L; FIB_min, minimum fibrinogen, g/L; PLT, platelet count, ×10^9^/L; INR, international normalized ratio; ALT, alanine aminotransferase, U/L; TB, total bilirubin, umol/L; LDH, lactate dehydrogenase,U/L; BUN, blood urea nitrogen, mmol/L; sCr, serum creatinine, umol/L; Lac, blood lactate, mmol/L.


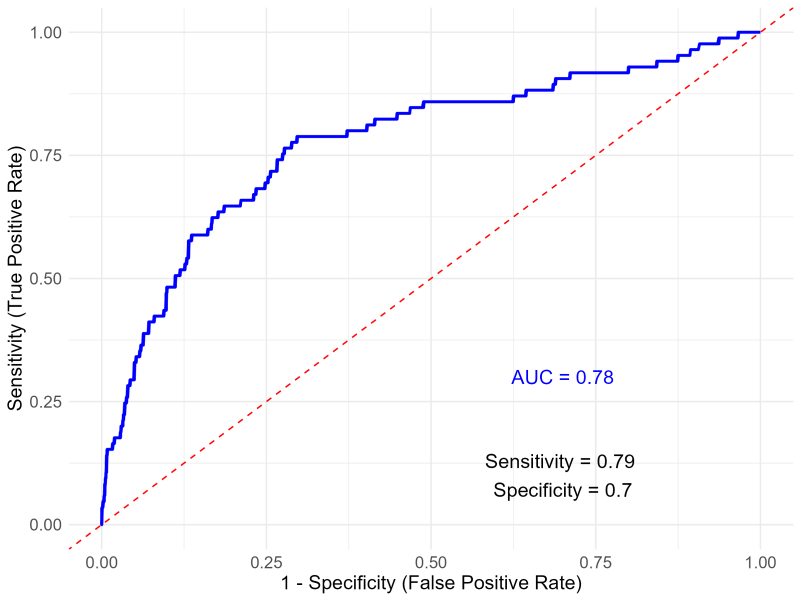


**Figure S3. ROC curve for the sepsis in-hospital mortality prediction model**


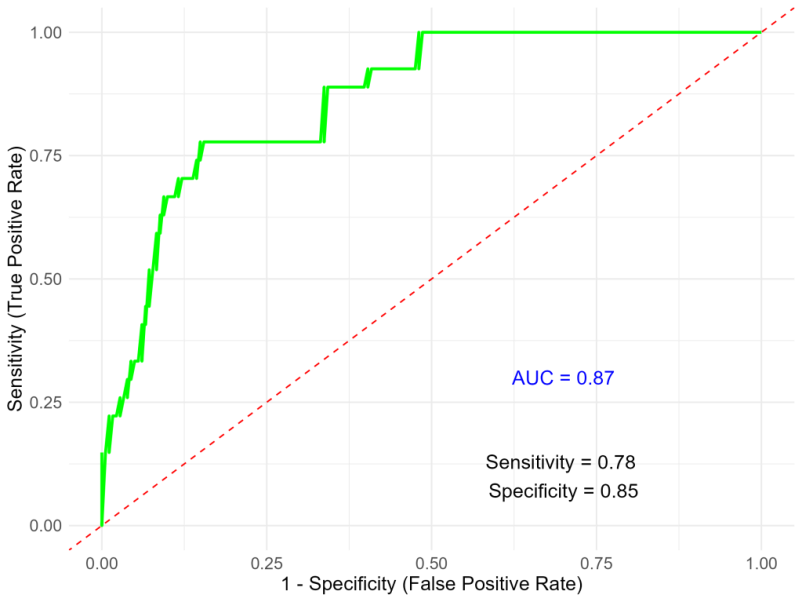


**Figure S4. ROC curve of the septic shock in-hospital mortality prediction model**

| **Table S1. Comparison of goodness-of-fit indices across trajectory models** | | | | | | | |
| --- | --- | --- | --- | --- | --- | --- | --- |
| **Group** | **Avepp(%)** | **OCC** | **P_j_(%)** | **π_j_(%)** | **BIC^#2^** | **△BIC^#2^** | **E_k_** |
| ***1Group (1 )*** | 100.00 |  | 100.00 | 100.00 | 7002.89 |  | 0.000 |
| ***2Group (2 2 )*** | 96.28-91.91 | 9.0-32.7 | 74.96-25.04 | 74.20-25.80 | 7986.58 | 983.69 | 0.828 |
| ***3Group (0 3 2 )*** | 87.42-89.40-93.03 | 11.0-7.4-151.6 | 38.96-53.31-7.72 | 38.81-53.10-8.09 | 8298.41 | 311.83 | 0.763 |
| ***4Group (1 1 1 1 )*** | 86.28-87.00-86.76-91.40 | 65.1-5.2-15.4-200.1 | 7.39-59.93-27.76-4.92 | 8.81-56.28-29.86-5.04 | 8417.01 | 118.60 | 0.779 |
| ***5Group (2 2 2 2 2 )*** | 92.97-85.20-88.21-81.55-91.70 | 607.5-9.3-9.4-47.0-154.9 | 2.04-38.46-45.42-7.47-6.62 | 2.13-38.32-44.31-8.59-6.66 | 8626.59 | 209.58 | 0.801 |

| **Table S2. Goodness-of-fit indices for the best-fitting group trajectory model** | | | | | |
| --- | --- | --- | --- | --- | --- |
| **Group** | **Class** | **OCC** | **Avepp(%)** | **Pj(%)** | **πj(%)** |
| **4Group** | Group1 | 65.1 | 86.28 | 7.39 | 8.81 |
|  | Group2 | 5.2 | 87.00 | 59.93 | 56.28 |
|  | Group3 | 15.4 | 86.76 | 27.76 | 29.86 |
|  | Group4 | 200.1 | 91.40 | 4.92 | 5.04 |

| **Table S3. Estimated parameters for the best-fitting group trajectory model** | | | | | | |
| --- | --- | --- | --- | --- | --- | --- |
| **Group** | **Class** | **Parameter** | **β** | **SE** | **t** | **P** |
| **4Group** | Group1 | Intercept | 0.58445 | 0.01474 | 39.651 | 0.00000 |
|  |  | Linear | 0.00158 | 0.00036 | 4.389 | 0.00001 |
|  | Group2 | Intercept | 0.75670 | 0.00534 | 141.704 | 0.00000 |
|  |  | Linear | -0.00066 | 0.00010 | -6.600 | 0.00000 |
|  | Group3 | Intercept | 0.84329 | 0.00648 | 130.137 | 0.00000 |
|  |  | Linear | -0.00034 | 0.00013 | -2.615 | 0.00892 |
|  | Group4 | Intercept | 1.00383 | 0.01215 | 82.620 | 0.00000 |
|  |  | Linear | -0.00085 | 0.00026 | -3.269 | 0.00108 |
| Note. Significance level: *P* ≤ 0.05. | | | | | | |

| **Table S4. Mathematical functions characterizing each trajectory subgroup** | |
| --- | --- |
| **Group** | **Mathematical function** |
| Group1 | ŷ= 0.58445 +0.00158x |
| Group2 | ŷ= 0.75670 -0.00066x |
| Group3 | ŷ= 0.84329 -0.00034x |
| Group4 | ŷ= 1.00383 -0.00085x |
| **Note: Logarithmic values were converted to blood glucose levels, e.g., 10^0.58445 = 3.8 (mmol/L), 10^0.75670 = 5.7 (mmol/L), 10^0.84329 = 7.0 (mmol/L), 10^1.00383 = 10.1 (mmol/L).** | |

| **Table S5. Univariate Logistic Regression Analysis of Risk Factors for In-Hospital Mortality in Sepsis Patients** | | | | | |
| --- | --- | --- | --- | --- | --- |
| **Variables** | **β** | **S.E** | **Z** | ***P*** | **OR (95%CI)** |
| **Age** |  |  |  |  |  |
| **≦1year** |  |  |  |  | 1.00 (Reference) |
| **>1year** | 0.26 | 0.24 | 1.09 | 0.275 | 1.29 (0.82 ~ 2.05) |
| **Sex** |  |  |  |  |  |
| **male** |  |  |  |  | 1.00 (Reference) |
| **female** | -0.21 | 0.23 | -0.88 | 0.379 | 0.81 (0.52 ~ 1.29) |
| **Trajectory phenotypes** |  |  |  |  |  |
| **Group2** |  |  |  |  | 1.00 (Reference) |
| **Group1** | 1.09 | 0.35 | 3.07 | **0.002^*^** | 2.98 (1.49 ~ 5.97) |
| **Group3** | 0.52 | 0.26 | 1.95 | **0.051*** | 1.67 (1.00 ~ 2.81) |
| **Group4** | 1.36 | 0.39 | 3.50 | **<.001*** | 3.88 (1.81 ~ 8.29) |
| **Septic shock** |  |  |  |  |  |
| **no** |  |  |  |  | 1.00 (Reference) |
| **yes** | 0.85 | 0.25 | 3.45 | **<.001*** | 2.35 (1.45 ~ 3.80) |
| **Mechanical ventilation** |  |  |  |  |  |
| **no** |  |  |  |  | 1.00 (Reference) |
| **yes** | 0.02 | 0.26 | 0.09 | 0.925 | 1.02 (0.62 ~ 1.70) |
| **Renal replacement therapy** |  |  |  |  |  |
| **no** |  |  |  |  | 1.00 (Reference) |
| **yes** | 1.89 | 0.24 | 7.83 | **<.001*** | 6.63 (4.13 ~ 10.65) |
| **PLT (×10^9^/L)** | -0.01 | 0.00 | -4.50 | **<.001*** | 0.99 (0.99 ~ 0.99) |
| **Hb (g/L)** | -0.02 | 0.01 | -2.98 | **0.003*** | 0.98 (0.97 ~ 0.99) |
| **CRP (mg/L)** | 0.00 | 0.00 | 0.89 | 0.376 | 1.00 (1.00 ~ 1.01) |
| **PCT (ng/ml)** | 0.00 | 0.00 | 0.38 | 0.705 | 1.00 (0.99 ~ 1.01) |
| **D-dimer (mg/L)** | 0.02 | 0.01 | 2.71 | **0.007*** | 1.02 (1.01 ~ 1.03) |
| **FIB_max (g/L)** | -0.08 | 0.06 | -1.22 | 0.223 | 0.93 (0.82 ~ 1.05) |
| **FIB_min (g/L)** | -0.26 | 0.08 | -3.16 | **0.002*** | 0.77 (0.66 ~ 0.91) |
| **INR** | 0.28 | 0.08 | 3.65 | **<.001*** | 1.32 (1.14 ~ 1.54) |
| **ALT (U/L)** | 0.00 | 0.00 | 1.74 | 0.083 | 1.00 (1.00 ~ 1.00) |
| **AST (U/L)** | 0.01 | 0.00 | 3.61 | **<.001*** | 1.01 (1.01 ~ 1.01) |
| **TB (umol/L)** | 0.01 | 0.00 | 5.76 | **<.001*** | 1.01 (1.01 ~ 1.01) |
| **ALB (g/L)** | -0.03 | 0.02 | -1.47 | 0.141 | 0.97 (0.94 ~ 1.01) |
| **BUN (mmol/L)** | 0.03 | 0.01 | 3.13 | **0.002*** | 1.03 (1.01 ~ 1.05) |
| **sCr (umol/L)** | 0.00 | 0.00 | 0.74 | 0.458 | 1.00 (1.00 ~ 1.00) |
| **Lac (mmol/L)** | 0.15 | 0.02 | 6.69 | **<.001*** | 1.16 (1.11 ~ 1.21) |
| OR, Odds Ratio; CI, Confidence Interval; PLT, platelet count; Hb, Haemoglobin; CRP, C-reactive protein; PCT, procalcitonin; FIB_max, maximum fibrinogen; FIB_min, minimum fibrinogen; INR, international normalized ratio; ALT, alanine aminotransferase; AST, aspartate aminotransferase; TB, total bilirubin; ALB, albumin; LDH, lactate dehydrogenase; BUN, blood urea nitrogen; sCr, serum creatinine; Lac, blood lactate; *Values marked in bold indicate statistical significance (*P* < 0.05). | | | | | |

| **Table S6. Multicollinearity Diagnostics for Variables in the Multivariate Regression Model** | | | |
| --- | --- | --- | --- |
| **Variables** | **GVIF** | **Df** | **GVIF^(1/(2*Df))** |
| **Trajectory phenotypes** | 1.082634 | 3 | 1.013321 |
| **Renal replacement therapy** | 1.186196 | 1 | 1.089126 |
| **Septic shock** | 1.015448 | 1 | 1.007694 |
| **Lac** | 1.164648 | 1 | 1.079189 |
| **TB** | 1.227120 | 1 | 1.107755 |

Lac, blood lactate;TB, total bilirubin.

| **Table S7. Univariate and Multivariate Logistic Regression Analysis of Risk Factors for In-Hospital Mortality in Septic Shock Patients** | | | | | | | | | | | |
| --- | --- | --- | --- | --- | --- | --- | --- | --- | --- | --- | --- |
| **Variables** | **Univariate** | | | | |  | **Multivariate** | | | | |
|  | **β** | **S.E** | **Z** | ***P*** | **OR (95%CI)** |  | **β** | **S.E** | **Z** | ***P*** | **aOR (95%CI)** |
| **VIS** |  |  |  |  |  |  |  |  |  |  |  |
| **0** |  |  |  |  | 1.00 (Reference) |  |  |  |  |  |  |
| **1** | -0.23 | 0.67 | -0.34 | 0.733 | 0.79 (0.21 ~ 2.97) |  |  |  |  |  |  |
| **2** | 0.96 | 0.51 | 1.90 | 0.057 | 2.62 (0.97 ~ 7.08) |  |  |  |  |  |  |
| **Trajectory phenotypes** |  |  |  |  |  |  |  |  |  |  |  |
| **Group2** |  |  |  |  | 1.00 (Reference) |  |  |  |  |  | 1.00 (Reference) |
| **Group1** | 2.47 | 0.60 | 4.12 | **<.001^*^** | 11.80 (3.65 ~ 38.15) |  | 1.65 | 0.72 | 2.29 | **0.022*** | 5.20 (1.27 ~ 21.28) |
| **Group3** | 0.92 | 0.53 | 1.74 | 0.082 | 2.51 (0.89 ~ 7.08) |  | 1.15 | 0.60 | 1.94 | 0.053 | 3.17 (0.99 ~ 10.18) |
| **Group4** | 2.00 | 0.80 | 2.51 | **0.012*** | 7.37 (1.55 ~ 35.09) |  | 2.11 | 0.88 | 2.40 | **0.016*** | 8.28 (1.47 ~ 46.55) |
| **Sex** |  |  |  |  |  |  |  |  |  |  |  |
| **male** |  |  |  |  | 1.00 (Reference) |  |  |  |  |  |  |
| **female** | -0.73 | 0.46 | -1.57 | 0.118 | 0.48 (0.19 ~ 1.20) |  |  |  |  |  |  |
| **Mechanical ventilation** |  |  |  |  |  |  |  |  |  |  |  |
| **no** |  |  |  |  | 1.00 (Reference) |  |  |  |  |  |  |
| **yes** | 0.16 | 0.53 | 0.30 | 0.767 | 1.17 (0.42 ~ 3.29) |  |  |  |  |  |  |
| **Renal replacement therapy** |  |  |  |  |  |  |  |  |  |  |  |
| **no** |  |  |  |  | 1.00 (Reference) |  |  |  |  |  | 1.00 (Reference) |
| **yes** | 2.43 | 0.46 | 5.27 | **<.001^*^** | 11.32 (4.59 ~ 27.89) |  | 1.54 | 0.56 | 2.74 | **0.006*** | 4.68 (1.55 ~ 14.12) |
| **PLT (×10^9^/L)** | -0.00 | 0.00 | -1.95 | 0.052 | 1.00 (0.99 ~ 1.00) |  |  |  |  |  |  |
| **WBC_min (×10^9^/L)** | -0.01 | 0.03 | -0.39 | 0.697 | 0.99 (0.92 ~ 1.05) |  |  |  |  |  |  |
| **WBC_max (×10^9^/L)** | -0.00 | 0.02 | -0.16 | 0.873 | 1.00 (0.96 ~ 1.03) |  |  |  |  |  |  |
| **Hb (g/L)** | -0.02 | 0.01 | -1.29 | 0.197 | 0.98 (0.96 ~ 1.01) |  |  |  |  |  |  |
| **CRP (mg/L)** | -0.01 | 0.00 | -1.50 | 0.134 | 0.99 (0.99 ~ 1.00) |  |  |  |  |  |  |
| **PCT (ng/ml)** | 0.00 | 0.01 | 0.57 | 0.569 | 1.00 (0.99 ~ 1.01) |  |  |  |  |  |  |
| **D-dimer (mg/L)** | 0.04 | 0.01 | 3.07 | **0.002*** | 1.04 (1.01 ~ 1.07) |  |  |  |  |  |  |
| **FIB_min (g/L)** | -0.40 | 0.16 | -2.41 | **0.016*** | 0.67 (0.49 ~ 0.93) |  |  |  |  |  |  |
| **ALT (U/L)** | 0.01 | 0.00 | 2.47 | **0.013*** | 1.01 (1.01 ~ 1.01) |  |  |  |  |  |  |
| **TB (umol/L)** | 0.01 | 0.00 | 3.02 | **0.003*** | 1.01 (1.01 ~ 1.01) |  |  |  |  |  |  |
| **ALB (g/L)** | 0.02 | 0.03 | 0.73 | 0.464 | 1.02 (0.96 ~ 1.09) |  |  |  |  |  |  |
| **BUN (mmol/L)** | 0.09 | 0.03 | 3.30 | **<.001*** | 1.09 (1.04 ~ 1.15) |  | 0.06 | 0.03 | 1.80 | 0.072 | 1.06 (0.99 ~ 1.13) |
| **sCr (umol/L)** | 0.00 | 0.00 | 1.05 | 0.292 | 1.00 (1.00 ~ 1.00) |  |  |  |  |  |  |
| **Lac (mmol/L)** | 0.15 | 0.04 | 3.98 | **<.001*** | 1.16 (1.08 ~ 1.25) |  | 0.10 | 0.05 | 2.03 | **0.042*** | 1.10 (1.01 ~ 1.21) |
| Note: VIS,Vasoactive-inotropic score; 0 = no vasoactive agents used (epinephrine, norepinephrine, dopamine, dobutamine, milrinone); 1 = only one vasoactive agent used; 2 = two or more vasoactive agents used; PLT, platelet count;WBC_min, minimum white blood cell count;WBC_max, maximum white blood cell count; Hb, Haemoglobin; CRP, C-reactive protein; PCT, procalcitonin;FIB_min, minimum fibrinogen; ALT, alanine aminotransferase; TB, total bilirubin; ALB, albumin; BUN, blood urea nitrogen; sCr, serum creatinine; Lac, blood lactate; .OR: Odds Ratio; aOR, adjusted Odds Ratio; CI: Confidence Interval.*Values marked in bold indicate statistical significance (*P* < 0.05). | | | | | | | | | | | |
